# Incidence of Post-Operative Pain following a Single-Visit Pulpectomy in Primary Molars Employing Adaptive, Rotary, and Manual Instrumentation: A Randomized Clinical Trial

**DOI:** 10.3390/medicina59020355

**Published:** 2023-02-13

**Authors:** Bhagyashree Thakur, Anuj Bhardwaj, Dian Agustin Wahjuningrum, Alexander Maniangat Luke, Krishna Prasad Shetty, Ajinkya M. Pawar, Rodolfo Reda, Marco Seracchiani, Alessio Zanza, Luca Testarelli

**Affiliations:** 1 Department of Dentistry, Division of District Early Intervention Centre, Thane Civil Hospital, Thane 400601, Maharashtra, India; 2 Department of Conservative Dentistry, Faculty of Dental Medicine, Universitas Airlangga, Surabaya City 60132, East Java, Indonesia; 3 Department of Conservative Dentistry and Endodontics, College of Dental Sciences & Hospital, Rau 453331, Indore, India; 4 Department of Clinical Science, College of Dentistry, Ajman University, Al-Jurf, Ajman P.O. Box 346, United Arab Emirates; 5 Centre of Medical and Bio-Allied Health Sciences Research, Ajman University, Al-Jurf, Ajman P.O. Box 346, United Arab Emirates; 6 Department of Conservative Dentistry and Endodontics, Nair Hospital Dental College, Mumbai 400008, Maharashtra, India; 7 Department of Oral and Maxillo-Facial Sciences, Sapienza University of Rome, Via Caserta 06, 00161 Rome, Italy

**Keywords:** adaptive instrumentation, postoperative pain, pulpectomy, randomized clinical trials, XP-endo Shaper

## Abstract

*Background and Objectives.* To differentiate the intensity of postoperative pain after primary molar pulpectomy employing manual instrumentation versus two single-file systems with different kinetics (the XP-Endo shaper file with adaptive instrumentation vs. the Kedo-SG blue file with continuous rotation instrumentation). *Materials and Methods*. This three-arm, single-blind, randomized clinical trial included assessing 75 healthy children between 4 to 9 years who required pulpectomy for primary molars (mandibular first and second). The three groups each had an equal number of children. Children in Group 1 had their teeth instrumented with the XP-endo Shaper, children in Group 2 had their teeth instrumented with the Kedo-SG Blue file, and children in Group 3 had their teeth instrumented manually using K-files. The degree of postoperative pain was measured using a four-point pain scale at 6-, 12-, 24-, 48-, and 72-h following therapy. Each participant’s parent received five flashcards with four faces and a word characterizing each face. The data were analyzed using Kruskal–Wallis and chi-square tests. The level of significance was set to 5%. *Results*. During the follow-up period, there was a significant difference in postoperative pain intensity between the three groups. The XP-endo shaper was associated with considerably decreased post operative at the 6- and 12-h interval followed by Kedo-SG. The highest post-operative discomfort across the groups was related to the patients who underwent manual instrumentation. *Conclusion*. In comparison to rotary and manual instrumentation, postoperative pain severity was reduced with adaptive instrumentation.

## 1. Introduction

In primary teeth with pulpal involvement, pulpectomy and root canal interventions are still the first choices of treatment. This intervention attempts to repair and/or preserve the affected periapical tissue, as well as to save the teeth until permanent successors emerge [1]. Removal of vital and necrotic pulp tissue, diseased dentine, and debris from the root canal system, as well as its abnormalities, should be the consequence of root canal instrumentation [2]. There has been significant interest in the development of pediatric endodontics, not only in terms of materials used but also in concepts of instrumentation methods.

Formerly, hand instrumentation was used for pulpectomy treatments. Barr et al. pioneered the use of motorized instrumentation for this practice, which was ascertained to be an accurate approach. The usage of motorized files shortened instrumentation time, which increased children’s compliance throughout therapy [3]. Every endodontic instrumentation approach transport debris further into the periapical tissues via the apical foramen. These debris might include necrotic pulp tissue, dentin chipping, and pathogens. Extrusion of this kind eventually results in postoperative discomfort and inflammation, as well as a slowing of periapical healing [4]. As an impact, the probable direct result of root canal intervention is pain or discomfort. The incidence of postoperative pain in children has been reported to be almost 66% [5]. Limiting pain is fundamental to the practice of pediatric dentistry since it can permanently scar young kids, jeopardizing their future dental procedures [5].

Thanks to the introduction of motorized nickel-titanium as the material of choice for the manufacturing of rotary instruments, root canal shaping has become more efficient, effective, and predictable, drastically reducing the operative time required both for permanent and primary root canal treatments [6,7,8]. Moreover, the development of heat treatments has remarkably increased the instruments’ mechanical performance both in terms of cyclic fatigue and torsional resistance, reducing the risk of their intracanal separation even in complex cases [9,10,11]. Nevertheless, the motorized files are insufficient for shaping wide and/or irregularly shaped root canals. They leave the majority of the root canal area largely untouched, eventually leaving diseased tissue intact. Root canals in primary molars are frequently ovoid or ribbon-shaped in the coronal portion and oval near the apex. Such canals present a significant difficulty for appropriate root canal cleaning, shaping, and disinfection, particularly when motorized endodontic tools are employed [12].

A novel adaptable motorized file design has been recently developed: the snake-shaped XP-endo Shaper file (FKG Dentaire, La Chaux-de-Fonds, Switzerland). This file system is intended to protect the peri-cervical dentin and maintain the natural canal architecture. The apparent MaxWire alloy (Martensite-Austenite Electropolishing-Flex, FKG), which responds at various temperatures, is used to manufacture this file. When the file is cooled, the martensite phase has an initial taper of 0.01; however, when it is exposed to body temperature (35 °C), the austenite phase’s molecular memory causes the taper to alter to 0.04 [13]. The file has been developed particularly to complement the 3D shape of root canal systems, including those with an oval cross-section. The “envelope of motion” of the file helps in restrictive dentin removal. In this technique the instrument builds a space reservoir with each envelope of motion, enabling the file to venture further toward the terminus of the root canal system [14]. With the XP-endo Shaper, the motion is flexible and can contract and expand as it moves down the length of the root canal. The file is said to have a six-blade tip labeled the Booster tip that enables it to shape the canal following a manual glide path of at least size 15 ISO and to steadily increase apical size to reach a size 30 ISO. An ultimate apical preparation of at least 30/0.04 is achieved by XPS [15,16]. The XP-endo Shaper’s design and operating minimizes stress on the dentine wall and provides outstanding debris evacuation, impressive flexibility, and cyclic fatigue resilience [17,18,19,20]. When utilized in non-round root canals, it has been shown to be more successful than solid metal-cored motorized files [17,18,19,20].

An increasing volume of research acknowledges the significance of minimizing post operative pain in pediatric endodontics using motorized instrumentation and specific pediatric rotary files [21,22,23,24,25]. Concerning to the Revo-S rotary system, Topçuoğlu et al. [21] found that canal preparation using hand files results in more severe postoperative discomfort. The author also cited the larger expulsion of debris by hand files compared to rotary Revo-S files as the cause of the hand files group’s higher pain threshold. According to Nair et al. [22], comparison of Kedo-S and M two rotary files with manual K-files, rotary file systems result in reduced pain frequency without significantly varying between the two rotary files. This was in line with research by Panchal et al. [23] that compared manual H- and K-files to Kedo-S rotary files. In all investigations, the maximum postoperative pain levels were recorded between 6 and 12 h afterwards, and it gradually reduced with time.

The degree of pain experienced after instrumenting the root canals of primary teeth with adaptive XP-endo Shaper files has not been documented to date. Hence, the objective of this study was to evaluate the degree of post-operative pain caused by a pediatric rotary and the innovative XP-endo Shaper files, both of which are powered by an endo-motor (motorized), to conventional hand-operated K-files. The null hypotheses tested in the current study was that the XP-endo Shaper will not differ concerning the post operative pain when compared to the contemporary pediatric rotary file Kedo-SG blue.

## 2. Materials and Methods

The current study was a three-arm, single-blind, randomized clinical trial executed at the College of Dental Science and Hospital, India and was approved by the institutional ethics committee (CDSH/IEC/05/2022; Approval date: 1 April 2022) and registered with the Clinical Trials Registry of India (CTRI/2022/04/041905) registered prospectively (19 April 2022). Comprehensive information regarding the required therapy was provided to the participant’s parents, and both verbal and written consents were acquired. A pilot study with 15 participants in each group was carried out as no previous research had been done implementing three groups to evaluate the post-operative pain. The findings of the pilot study Odd’s ratio of 6.5 between XP endo and Hand file and to achieve a power of 0.80 at a 0.05 level of significance and a minimum of 24 had to be evaluated but we rounded it off to 25. The G*Power (Version 3.1.9.3; Heinrich Heine University, Düsseldorf, Germany) was used to estimate the sample size. Hence, the sample size for the main investigation was 25 individuals in each group. The post-operative pain score was considered for sample size estimation as we have also recorded the pain score as ordinal data.

### 2.1. Patient Recruitment and Allocation

**Figure 1:**
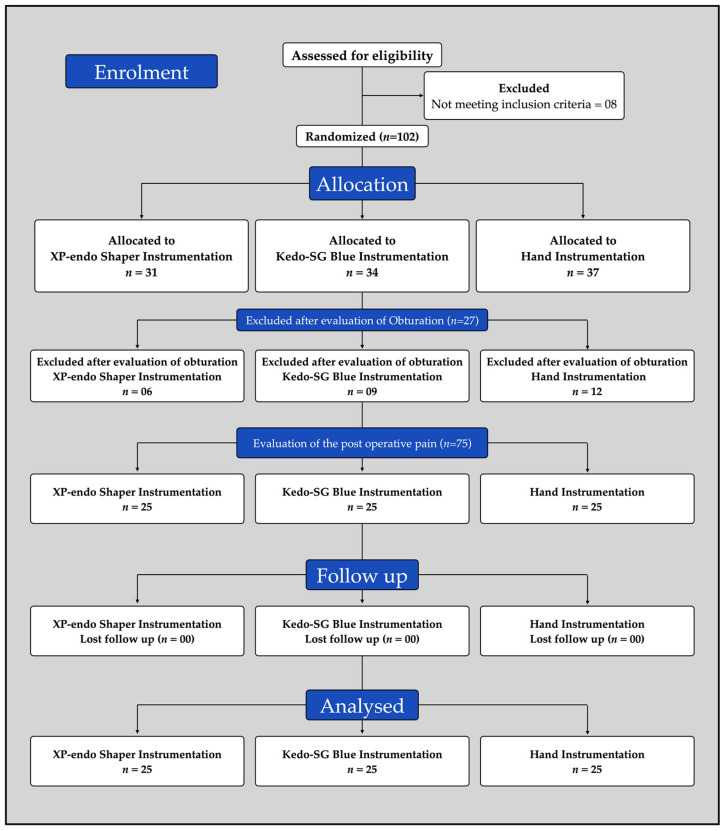
CONSORT 2010 flow diagram.

**Figure 2:**
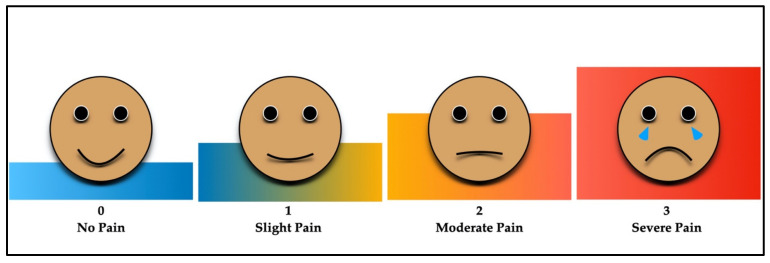
Four-point facial pan intensity scoring system.

The current study was carried out in accordance with the Consolidated Standards of Reporting Trials (CONSORT) group’s standards for clinical trial planning and reporting (Figure 1). The study included children of both genders and ages ranging from 4 to 9 years, who needed pulpectomy in their primary mandibular molars (first “D” and second “E”). A total of 110 patients were enrolled after acquiring informed consent from the accompanying parent. Prior to performing the pulpectomy, a four-point pain scale was employed to quantify the pre-instrumentation pain score [26]. The following four-point scale was used to assess pain: (1) zero—no pain, (2) one—slight pain, (3) two—moderate pain, and (4) three—severe pain (Figure 2) as reported by Topçuoğlu et al. [27]. The investigation involved radiographic examinations performed prior to treatment that revealed no interradicular radiolucency or periapical lesions. In addition, only teeth with three root canals following access cavity preparation were included in the trial to standardize the groups.

Acute apical periodontitis, necrotic pulp, more than three root canals, and individuals with abscess were disqualified. Patients who took medications up to six hours before the treatment were also barred from participating in the study. Children with a lack of cooperative capacity, those with a systemic ailment, or those with particular healthcare needs were also excluded from the study. A total of 8 individuals were disqualified because they did not match the inclusion criteria.

The block randomization strategy was employed to divide the individuals *n* = 31, 34, and 37 for XP-endo Shaper, Kedo-SG Blue, and hand file groups, respectively. A statistician created the randomized sequence, and the allocation was hidden by using opaque envelopes. The treatment technique was kept undisclosed from the patients and their parents, and the evaluator who recorded the instrumentation time was likewise blinded. The operator (B.T.) was delivering the therapy, hence the operator could not have been rendered blind.

The pulpectomy treatment was executed by the same operator (B.T.) and completed in a single visit. Non-pharmacological behavior management approaches were employed to change the child’s attitude and seek cooperation. Local anesthetic (2% lignocaine and 1:200,000 adrenaline; LIGNOX, Indoco Remedies Limited, Mumbai, India) inferior alveolar (I.A.) nerve block that enables a reasonably long-lasting anesthesia was performed, proceeded by rubber dam isolation of the tooth (CricDental Rubber Dam Kit, Mumbai, India). This was continued by caries removal and access cavity creation utilizing a high-speed no.4 round diamond point (Dentsply Maillefer, Tulsa, OK, USA). The root canal orifices were then recognized using the DG-16 explorer (Hu-Friedy, Chicago, IL, USA) only after de-roofing the entire pulp chamber. For standardized groups, teeth with only 3 root canal orifices were included in the current clinical trial. The root canal patency was checked for all the canals located using a size #10 (0.02%) k-file (NiTi flex; Dentsply Sirona, Charlotte, NC, USA) followed by estimation of the working length (WL) as 1 mm short of the apex using an electronic apex locator (ProPex Pixi; Dentsply Sirona, Charlotte, NC, USA) and the root canal instrumentation was initiated.

The randomization procedure was used to select the kind of instrumentation for the specific tooth. The adaptive XP-endo Shaper (tip size #30; FKG Dentaire, La Chaux-de-Fonds, Switzerland) was used for root canal instrumentation in Group 1; for Groups 2 and 3, Kedo-SG Blue pediatric rotary files (E1 tip size #0.30; Reeganz Dental Care Private Limited, Chennai, India) and a hand K-file (Dentsply Maillefer, Tulsa, OK, USA) up to #30 were used, respectively. The rotary files were powered with the aid of X-smart Plus endo-motor (Dentsply Sirona, Charlotte, NC, USA) according to the manufacturer’s instructions. The XP-endo Shaper was operated at 800 rpm and 1 Ncm torque, while the Kedo-S file was used at 250 rpm and 2.2 Ncm torque.

### 2.2. Root Canal Instrumentation

#### 2.2.1. XP-Endo Shaper Instrumentation

Root canal instrumentation was performed on the assigned patients utilizing 21 mm XP-endo Shaper files. After access cavity preparation, the canal patency was inspected using a #10 K-file, and if desired, a glide path until a #15 K-file was prepared. The root canals were loaded with 2 mL of 2.5% NaOCl before the XP-endo Shaper file was inserted passively until resistance was reached. After retracting the tip coronally, the endomotor was powered at 800 rpm and 1 Ncm torque, and the file was reintroduced. The file was used 4–5 times toward the WL with delicate vertical strokes. When the WL was achieved, the file was retracted and cleaned, apical patency was ascertained with the #15 K-file, the canal was irrigated again with 4 mL of 2.5% NaOCl, and the file was used for an additional 15 thrusts to the WL, designating instrumentation fulfilment, accompanied by irrigating the canal with 4 mL of 2.5% NaOCl, as per manufacturer’s instructions. Eventually, the canals were irrigated with 2 mL of 17% EDTA and normal saline.

#### 2.2.2. Kedo-SG Blue Instrumentation

Kedo-SG Blue was used for root canal instrumentation on the patients in this group. It is a one file system, and the E1 file employed in this study has a #30 tip with an alternate taper of 0.04 to 0.08. (Reeganz Dental Care, Chennai, India). These are 16-mm files that were driven by an electronic endomotor at 250 rpm and 2 Ncm torque (X-Smart Plus; Dentsply Maillefer, Ballaigues, Switzerland). There was no pre-flaring done. Until the WL was achieved, a permissive alternating apico-coronal motion was implemented. The irrigation protocol was performed similarly as reported in the first group.

#### 2.2.3. Hand Instrumentation

The quarter-turn-and-pull action was used to instrument the root canals of the subjects in this group. Hand K-files #15/0.02, #20/0.02, #25/0.02, and #30/0.02 were navigated in a series of stainless-steel K-files (Mani, Tokyo, Japan). The irrigation protocol was performed similarly as reported in the first group.

### 2.3. Root Canal Obturation

The canals were dried with #30 paper points, a 0.04 taper for Groups 1 and 2, and a 0.02 taper for Group 3, after root canal instrumentation and final irrigation. Using a pressure syringe with a needle, the root canals were then filled with an iodoform-based calcium hydroxide paste (Metapex, META Biomed, Colmar, PA, USA). Additionally, the paste was pushed to just short of the apex with the hand file. The excess paste was scraped from the coronal cavity, which was then filled with glass ionomer cement (Vitrebond, 3M ESPE, St Paul, MN, USA). Finally, on the same visit, a preformed metallic crown (3M ESPE) was selected, high points were adjusted by assessing the occlusion with articulating paper and finally cemented (PCA, SS White, Gloucester, UK).

### 2.4. Post-Obturation Assessment

Based on the criteria provided by Sandrian and Coll, the obturation quality was assessed radiographically [26]. The criteria were (i) Under filling: Every canal had filling that was more than 2 mm short of the radiography apex; (ii) Optimal filling: Obturation material may end in one or more canals till the radiographic apex or within up to 2 mm short of the apex; and (iii) Over filling: Any of canal that exhibits obturation beyond the radiographic apex. Only teeth exhibiting optimal obturation were included in the study.

### 2.5. Post-Operative Pain Assessment

The modified Wong–Baker pain rating scale was utilized to ascertain the postoperative pain. Each participant’s parent was given a questionnaire to answer in order to assess their child’s level of discomfort following the pulpectomy procedure at intervals of 6, 12, 24, 48, and 72 h. A pain-intensity scale was illustrated to each participant and their parents by an outcome assessor who was unaware of the research groups. The outcome assessor, who was blinded to the research groups, also called the participant’s parent to record the severity of the postoperative pain in order to prevent bias. This was done at various intervals. During the second appointment, the questionnaire was also acquired from the participant’s parent(s), and the outcome assessor cross-checked it to ensure that the values reported were consistent. The treatment strategy for the children was kept hidden from the parents taking part in the trial. All participants were prescribed ibuprofen (or, if contraindicated, paracetamol) to be administered in the event of severe pain. The pain was recorded using a 4-point pain scale (0—absence of pain; 1—minimal pain; 2—moderate pain; and 3—severe pain) as previously reported [27].

### 2.6. Statistical Analysis

The acquired data were entered into a spreadsheet, and statistical analysis was performed using SPSS software version 22 (IBM Corp, Armont, NY, USA). Demographic data and the pre-operative pain score were compared between the groups using the Chi-square test of proportion for the categorical and Analysis of Variance for the continuous variable. To compare the pre-operative pain score between the groups, the Kruskal–Wallis test was applied. To compare post-operative pain within the group at different time intervals, the Chi square test of proportion was done and for intergroup comparison, the Kruskal–Wallis test was conducted. All statistical analyses were done at the 95% Confidence Interval and *p* value less than 0.05 was considered statistically significant.

## 3. Results

A total of 27 teeth of the 102 teeth that underwent pulpectomies, with different root canals instrumentations (06 for XP-endo; 09 for KedoSG-blue; and 12 for hand k-file), were excluded from evaluation as they were associated with the quality of obturation being either under-filled or over-filled. Consequently, 75 teeth (*n* = 25 per group) that exhibited optimal obturation were assessed for post-operative pain. Of the 75 children included, 36 were girls and 39 were boys. The mean ages, tooth included, and the pre-operative pain are presented in Table 1. Concerning the baseline variables, no statistically significant difference was seen in the study population between the groups (Table 1).

Table 2 shows the post-operative pain intensities for the three groups at various time points. When the pain score was compared within the group, there was a statistical significant difference in proportion seen in the Hand K-file group at 6-, 12-, and 24-h intervals (*p* < 0.05). At the 12-h interval, significant difference in proportion was also seen in XP-endo Shaper and Kedo-SG Blue groups (*p* < 0.05). There was a statistically significant disparity between the groups at 6 h (*p* = 0.009) and 12 h (0.023). The severity of pain reported at 6- and 12-h intervals was greater in patients who received root canal instrumentation with the rotary pediatric Kedo-SG Blue and manual k-files compared to the XP-endo Shaper adaptive file group.

When teeth were instrumented with a high-speed XP-endo Shaper, the pain subsided over a period of 6 h, whereas it subsided over a time of 12 h and subsided over a period of 24 h for teeth that were instrumented with rotary Kedo-SG blue and manual k-files.

The teeth instrumented using high-speed XP-endo Shaper files exhibited least post-operative pain at 6 h and 12 h compared to the other files investigated (*p* < 0.05). At 12 h, there was no significant difference between post-operative pain after instrumentation with Kedo-SG blue and manual k-file (*p* > 0.05). At 24-, 48-, and 72-h intervals, there was no significant difference in the post-operative pain between three groups (*p* > 0.05).

The frequency of children who needed analgesics was two (8%) in the XP-endo Shaper group, four (16%) in the Kedo-SG Blue group, and five (20%) in the Hand K-file group.

## 4. Discussion

Post-operative pain has a significant impact on the clinical efficacy of endodontic therapy in both primary and permanent dentition. In young children, enhanced post-operative agony is typically associated with increased anxiety. The goal of endodontic therapy should be to provide optimal treatment with minimum post-operative pain [28]. Analgesics are frequently used to relieve post-operative pain. Nonsteroidal anti-inflammatory drugs and opioids are commonly used to alleviate post-operative endodontic pain [29]. Multiple analgesic dosages, on the other hand, have been associated to deleterious effects in children, such as respiratory depression, sleepiness, nausea, and vomiting [30].

Dentinal remnants, pulp tissue, necrotic detritus, irrigation fluids, and microorganisms are all imperceptibly pushed into the periapical tissues during bio-mechanical preparation. In light of physiological root resorption, primary teeth may exhibit increased apical extrusion of debris. Expelling these substances may have unintended consequences, such as inflammation, sluggish healing, and surgical pain [31]. As a result, the present randomized clinical research, which employed three distinct endodontic instrumentation techniques, was focused on post-operative pain following one-visit pulpectomy therapy.

For root canal instrumentation in this study, an adaptive XP-endo Shaper, a pediatric rotary Kedo-SG blue, and traditional manual instrumentation with K-files were used. The file systems were compared, and each was operated in accordance with the manufacturer’s specifications. As a result, there was no attempt to artificially equalize characteristics such as the number of instruments used or the time necessary to finish the treatment.

Motorized instrumentation using Ni–Ti files has been shown to minimize working time in pediatric endodontics [3,32,33]. Additionally, these files are reported to be associated with reduced post-operative pain [34,35], which may be attributed to the minimized extrusion of debris during instrumentation by the rotary files [36].

The Kedo-SG blue pediatric nickel-titanium (NiTi) rotary file (Reeganz Dental Care, Chennai, India) is a single file system for root canal instrumentation. The better results in reduced post-operative pain in this group could be attributed to minimal apical extrusion of debris due to the file’s variably variable taper, specifically designed tip diameter, and the fact that these files are manufactured from a metal that is exported to blue heat treatment [15,16,28,33,37]. This thermal treatment aids in increasing the flexibility and reducing the microhardness of the files. These properties may cause reduced debris extrusion beyond the apex, thus reducing post operative pain [37]. An optimal obturation in primary teeth may also result in reduced post-operative pain [24].

Canal preparation using hand files, according to Topçuoğlu et al. [27], induces more significant postoperative discomfort than the tested rotary file. Nair et al. [22] assessed Kedo-S and Mtwo rotary files to manual K-files and found that the rotary file system had reduced pain frequency with no variation between the two rotary files. This was consistent with the findings of Panchal et al. [23], who compared the Kedo-S rotary file to manual H- and K-files. In all investigations, the maximum postoperative pain levels were recorded at 6–12-h intervals and reduced with time. This is consistent with the results of the current investigation, which indicated that rotary instrumentation was significantly associated to less post-operative discomfort than manual instrumentation. A recent investigation by Elheeny and Abdelmotelb showed that both rotary and reciprocating files did not differ in the post operative pain when employed for root canal instrumentation in primary teeth [38].

Metapex, an iodoform-based calcium hydroxide cement, is the most commonly used obturating material during pulpectomy [39]. When used for obturation, this cement has the benefit of showing no foreign body response when extruded into furcal or apical locations. There have been no reports of the extruded Metapex having any impact on the bud stages of permanent teeth [40]. Uncontrolled root canal instrumentation causes apical ejection of the obturating material, resulting in the release of iodoform, a potent irritant, into the periapical region, triggering an inflammatory reaction that causes discomfort [33]. The pediatric rotary file is reported to be associated with significantly optimal obturation compared to manual instrumentation [1]. This could be the possible reason for less post-operative pain in the teeth that were instrumented using rotary Kedo-SG blue files compared to manual instrumentation.

The root canals in primary teeth are large and irregularly shaped in cross-section, which pose difficulty while attempting to fulfill and optimum root canal instrumentation [41]. The employment of solid core rotary files usually leads to a circular preparation, leaving more than half of the root canal perimeter not only unaltered but also resulting in the removal of amounts of radicular dentin and packing them either laterally or apically [12,42].

The adaptive shaping concept was developed to address this problem by the introduction of files with expanding cores addressing to the irregular shapes of the root canals [16]. The XP-endo Shaper (snake-shaped) is one such file which has been extensively researched and found superior and associated with reduced apical debris extrusion, increased intra-canal bacterial reduction, and least post-operative pain, especially in non-round root canals of adult teeth [1,28,33,43,44]. These files were also reported with reduced debris apically and significantly optimum obturation in primary teeth [1]. The reduced debris in this group could be because of the presence of loose space around the file. Another factor that might explain the discomfort disparities between the studied systems is the quantity of debris left in the root canal. While rotating at 800 rpm and 37 °C, the file features a 0.04 taper envelope of movement with a hollow center. Hence, the debris are suspended and carried coronally with a tornado-like movement of the irrigant created by the speed of rotation (800 rpm) and the snake-like shape of the file is more efficient than pushing debris coronally, as in the case of rotary files [1,28,33,43,44]. The post-operative pain after single-visit pulpectomy was significantly less with the XP-endo Shaper file compared to the other two groups evaluated at all intervals. As a result, the null hypothesis was disproved. To the best of the authors’ awareness, the effectiveness of this file on post-operative pain in primary teeth is yet to be reported in literature.

The current study evaluates pain using a subjective technique. This is the prime bias-creating limitation in the current study and earlier studies examining post-operative pain. In addition, the relatively low sample size and the fact that the quality of obturation was only evaluated using 2D periapical radiographs were other potential drawbacks of the current investigation. In pediatric clinical research, however, 2D periapical radiographs was the sole option.

## 5. Conclusions

Within the limitations of the study, it can be concluded that the patients in which XP-endo Shaper files was employed for root canal instrumentation were associated with least post-operative pain after single-visit pulpectomy in their primary molars, followed by Kedo-SG blue and manual instrumentation.

## Figures and Tables

**Table 1:** Demographic details and preoperative score distinctions between three groups.

	XP-Endo Shaper	Kedo-SG Blue	Hand K-File	*p* Value
**Gender**				
Girls ^$^	11	13	12	1.00
Boys ^$^	14	12	13	1.00
Age ^#^	5.37 ± 0.83	5.61 ± 0.75	5.43 ± 0.71	0.54
Mandibular molar “D” ^$^	12	14	11	1.00
Mandibular molar “E” ^$^	13	11	14	1.00
Pre-operative pain score ^£^				
No Pain	9	8	9	
Slight	10	10	11	0.854
Moderate	6	7	5	
Severe	0	0	0	

*p* < 0.05 denotes statistically significant difference. All the values exhibit non-significant difference. Statistical test applied, ^$^ Chi-square test of proportion, ^#^ Analysis of Variance ^£^ Kruskal–Wallis.

**Table 2:** The intensity of postoperative pain at different points in time in the groups.

Time Periods	Pain Score	XP-Endo Shaper	Kedo-SG Blue	Hand K-File	*p* Value ^$^
**6 h**	No Pain	11	9	3	0.009 *
	Slight	9	8	11	
	Moderate	5	6	9	
	Severe	0	2	2	
	*p* value ^#^	0.326	0.204	0.024 *	
**12 h**	No Pain	23	17	13	0.023 *
	Slight	2	5	9	
	Moderate	0	3	3	
	Severe	0	0	0	
	*p* value ^#^	0.000 *	0.001 *	0.048 *	
**24 h**	No Pain	25	25	23	0.37
	Slight	0	0	2	
	Moderate	0	0	0	
	Severe	0	0	0	
	*p* value ^#^	-	-	0.000 *	
**48 h**	No Pain	25	25	25	1
	Slight	0	0	0	
	Moderate	0	0	0	
	Severe	0	0	0	
	*p* value ^#^	-	-	-	
**72 h**	No Pain	25	25	25	1
	Slight	0	0	0	
	Moderate	0	0	0	
	Severe	0	0	0	
	*p* value ^#^	-	-	-	

* *p* < 0.05 denotes statistically significant difference. Statistical test applied ^#^ Chi square test of proportion, ^$^ Kruskal–Wallis.

## Data Availability

The data presented in this study are available on request from the corresponding author.
